# Comparative efficacy of olaparib in combination with or without novel antiandrogens for treating metastatic castration-resistant prostate cancer

**DOI:** 10.3389/fendo.2023.1225033

**Published:** 2023-10-31

**Authors:** Xiangyu Chen, Yang Pan, Qihua Wang, Congzhe Ren, Muwei Li, Xuexue Hao, Lijun Xie, Xiaoqiang Liu

**Affiliations:** Department of Urology, Tianjin Medical University General Hospital, Tianjin, China

**Keywords:** metastatic castration-resistant prostate cancer, novel antiandrogens, the poly(ADP-ribose) polymerase inhibitor, olaparib, monotherapy, combination therapy

## Abstract

**Background:**

Studies using novel antiandrogens (NAA) in patients with metastatic castration-resistant prostate cancer (mCRPC) have shown overall survival benefit. As patients develop resistance to NAA therapy, the poly(ADP-ribose) polymerase inhibitor (PARPi) olaparib in combination with NAA may become a promising therapy. However the overall benefit of olaparib monotherapy or combination therapy still needs to be evaluated. Therefore, we performed a network meta-analysis to assess the efficacy and toxicity between olaparib, olaparib combined with abiraterone and NAA.

**Methods:**

We searched PubMed, EMBASE, the Cochrane Library and American Society of Clinical Oncology (ASCO) University Meeting abstracts for randomized controlled trials reporting olaparib and NAA from 2010 up to March, 2023. Network meta-analysis using Stata 16.0 and R 4.4.2, hazard ratios (HR) with 95% confidence intervals (CI) were used to assess the results.

**Results:**

Four trials reported olaparib, olaparib plus abiraterone and apalutamide plus abiraterone. radiographic progression-free survival (rPFS) was significantly lower in patients on apalutamide plus abiraterone compared to olaparib (HR, 1.43; 95% CI, 1.06-1.93). rPFS was similar for olaparib plus abiraterone and olaparib (HR, 1.35; 95% CI, 0.99-1.84); likewise, olaparib plus abiraterone and apalutamide plus abiraterone were similar (HR, 1.06; 95% CI, 0.83-1.35). In addition, there was no significant difference between the three interventions for OS. But olaparib has the highest probability of being a preferred treatment for improving rPFS and OS.

**Conclusion:**

rPFS was in favor of olaparib compared with apalutamide plus abiraterone. But there were no difference between olaparib plus abiraterone and either olaparib or apalutamide plus abiraterone. Apalutamide plus abiraterone might be the most preferred intervention in cases where AEs are involved.

**Systematic review registration:**

https://inplasy.com, identifier INPLASY2023100072.

## Introduction

Prostate cancer is the second most common cancer after lung cancer and accounts for 7% of all new cancer diagnoses in men worldwide (1, 2). The standard of care for metastatic castration-resistant prostate cancer (mCRPC) includes taxane-based chemotherapy, radiotherapy, and novel antiandrogens (NAA), such as abiraterone, enzalutamide, darolutamide, or apalutamide. Although these treatment options have shown the ability to improve overall survival (OS) (3–6), subsequent NAA therapy is known to work only in a minority of patients and the responses are short-lived (7, 8). There is an urgent need to evaluate non-NAA monotherapy approaches in light of the growing number of patients receiving their first NAA therapy before developing castration-resistant disease.

Notably, 15-30% of patients with mCRPC have mutations in homologous recombination repair (HRR) genes (including BRCA1, BRCA2, ATM, and CHEK2) (9, 10). Olaparib has been recently approved by the U.S. Food and Drug Administration (FDA) as a potent orally bioavailable poly(ADP-ribose) polymerase (PARP) inhibitor to treat BRCA-1/2-deficient patients (11). It possesses both *in vitro* cellular potency and *in vivo* efficacy, and it led to 80% tumor inhibition when administered in combination with temozolomide in mice (12). In a preliminary phase II study, patients with mCRPC who previously received NAA treatment were administered olaparib. Tumors with HRR mutations showed more positive responses to olaparib than those without mutations (13). In preclinical studies, olaparib synergizes with agents that affect the androgen receptor (AR) pathway regardless of the HRR mutation status (14). As patients develop resistance to NAA treatment, understanding the mechanism by which co-inhibition of AR and PARP proteins leads to the inability of cancer cells to repair DNA may facilitate the use of a PARP inhibitor (PARPi) in combination with NAA. However, the overall benefits of olaparib monotherapy and combination therapy still need to be evaluated. Therefore, we performed indirect comparisons and network meta-analyses to assess the efficacy and toxicity of olaparib, olaparib combined with abiraterone, and NAA to provide a basis for clinical drug selection for patients with mCRPC.

## Methods

### Search strategy and study selection

**Figure 1:**
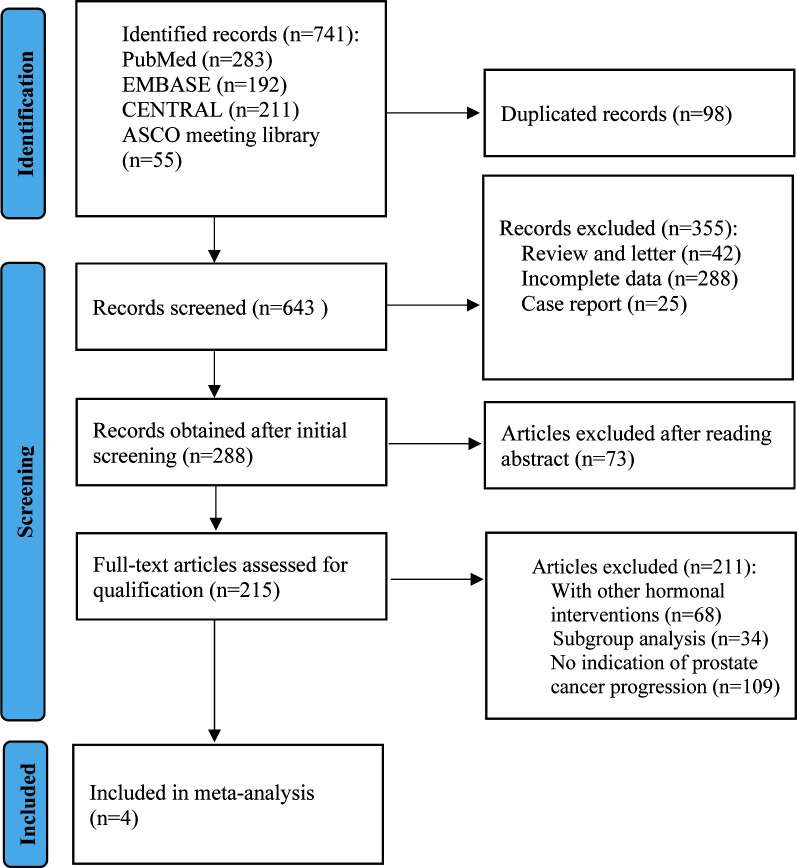
Flow chart for PRISMA-based articles screening.

The PRISMA guidelines were followed in this systematic review and meta-analysis was being conducted (15). As detailed in the PRISMA flow chart (**Figure 1**), we reviewed PubMed, EMBASE, the Cochrane Central Register of Controlled Trials (CENTRAL) (The Cochrane Library), and American Society of Clinical Oncology (ASCO) University Meeting abstracts for citations from 2010 to March 2023. The search criteria were limited to articles published in English and phase II or phase III RCTs on patients with mCRPC. The full search strategy and inclusion–exclusion criteria are outlined in **Supplementary Material**. We used the most recent or comprehensive study for studies in different journals with overlapping data, duplicate data, or the same authors.

### Inclusion and exclusion criteria

The inclusion criteria for eligible studies were as follows: (a) randomized controlled design; (b) inclusion of only mCRPC patients; (c) provision of at least one of the following oncologic outcomes: radiologic progression-free survival (rPFS) or OS; (d) inclusion of primary and secondary endpoints; and (e) extraction of either the hazard ratio (HR) or the number of events from the text; (f) Interventions were limited to treatment line I or II. The exclusion criteria were as follows: (a) publications that were duplicated or contained poor-quality information; (b) studies that contained insufficient primary data or incomplete study data; and (c) publications that were reviews, commentaries, letters, or case reports.

### Data extraction and study quality

**Figure 2:**
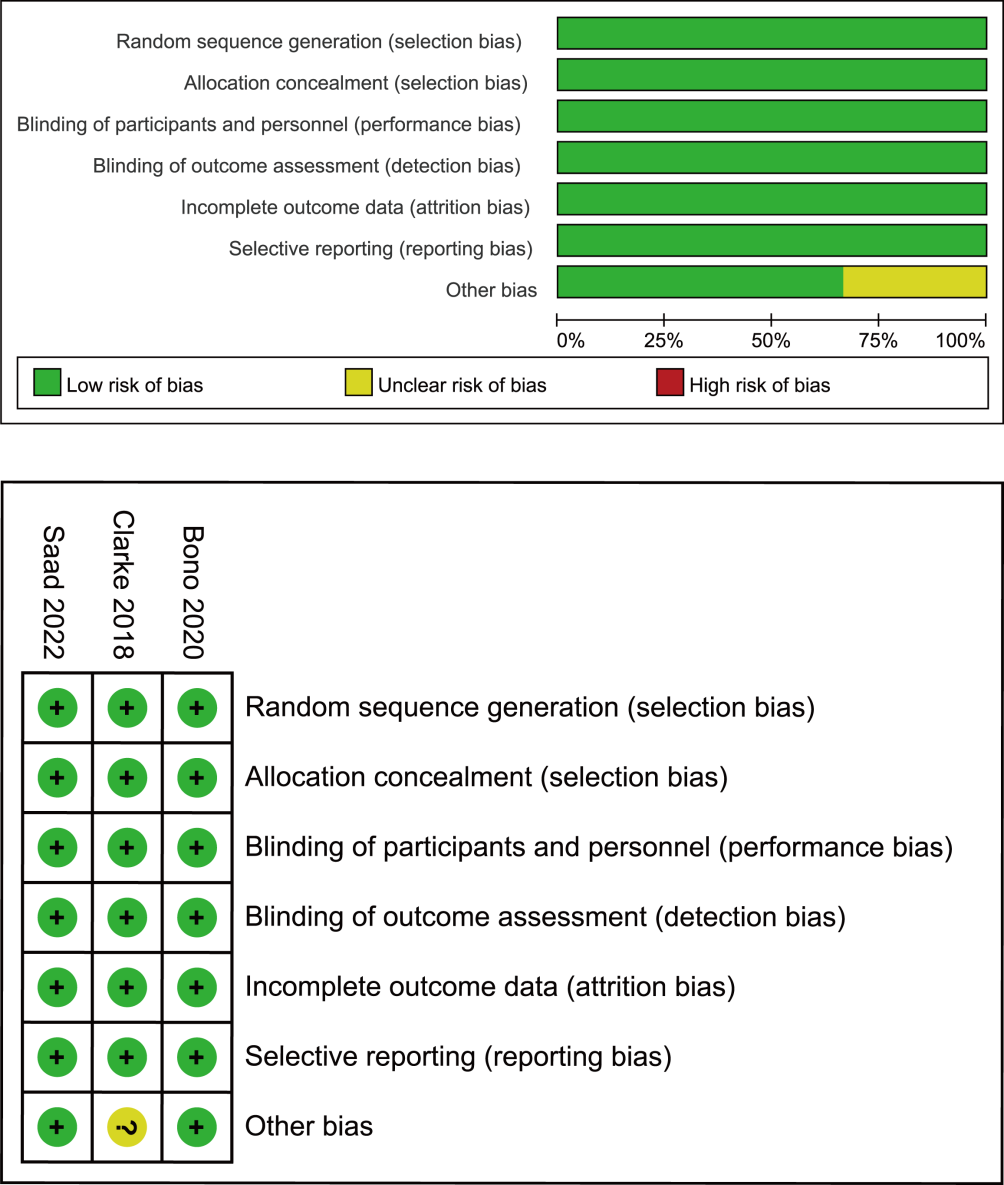
Bias risk assessment criteria for randomized controlled trials based on the Cochrane Collaborative Network.

First, two researchers independently screened the literature and extracted data according to established criteria. The reasons for excluding the articles were also recorded. When a disagreement arose, both parties negotiated with or consulted a third-party expert. Records included the first author, year of publication, clinical trial name, cancer characteristics, median age, interventions, median levels of prostate-specific antigen (PSA), and Eastern Cooperative Oncology Group. For each study, HR, the calculated odds ratio (OR) and confidence intervals (CI) were extracted for the reported primary and secondary endpoints, which included rPFS, OS, time to second progression-free survival (PFS2; defined as time from randomization to the investigator-assessed progression event [using Response Evaluation Criteria In Solid Tumors (RECIST) version 1.1 or Prostate Cancer Clinical Trials Working Group 2 (PCWG2) criteria] following that used for the primary rPFS analysis, or death), objective response rate (ORR; RECIST v 1.1, PCWG2) (16), PSA response (reduction of ≥50% from baseline, confirmed at the next assessment ≥4 weeks later), circulating tumor cell (CTC) conversion (change from ≥5 cells/7.5 mL at baseline to <5 cells/7.5 mL post-baseline). We also extracted the number of overall adverse events (AEs) and noted the number of severe adverse events (grade ≥3). The quality of the included trials was assessed using the Cochrane Collaboration tool to assess the risk of bias in the randomized controlled trials (**Figure 2**) (17).

### Statistical analysis

The data were processed using Stata 16.0 and R 4.4.2. A Bayesian network meta-analysis was used for indirect comparisons of selected endpoints using the GeMTC package in R. We used the reported HR or calculated OR in the analysis. Considering that there was only one point of data for each intervention, no source of inconsistency was assessed; therefore, indirect comparisons between different interventions were obtained using a fixed-effects model. We used the rank probabilities for the primary and secondary endpoints to assess the preferred probability ranking for each drug.

## Results

### Characteristics of included studies

The electronic search revealed 741 citations; after screening, 737 records were eliminated because they did not meet the initial requirements (**Figure 1**). At the end of the review process, four studies were included in the qualitative and quantitative syntheses (18–21). A total of 2307 patients from all four studies were assigned to receive either olaparib, olaparib plus abiraterone, apalutamide or a placebo. The characteristics of the included studies are summarized in **Table 1**. Patients with prostate cancer had been diagnosed by cytology and histology, and metastases were detected by bone scanning, contrast-enhanced computed tomography, or magnetic resonance imaging. **Table 2** summarizes the results for each study endpoint, for which the investigators graded the AEs according to the National Cancer Institute Common Terminology Criteria for Adverse Events. **Table 3** summarizes the results of indirect comparisons of three interventions in each endpoint. Quality of the included studies was assessed (**Figure 2**).

**Table 1:** Characteristics of the trails included in the meta-analysis.

First author	Year	Clinical trial	Median age (yr)	Intervention arm	Control arm	Median PSA (μg/L or ng/mL)	ECOG performance status	Treatment line^b^
Bono (19)	2020	PROfound	69	Olaparib	Abiraterone or enzalutamide	68.2^a^	0(51%) 1(44%) 2(5%)	II
Clarke (18)	2018	NCT01972217	70	Olaparib plus abiraterone	Placebo plus abiraterone	86.0^a^	0(48%) 1(51%) 2(1%)	I/II^c^
Saad (21)	2022	PROpel	—	Olaparib plus abiraterone	Placebo plus abiraterone	—	—	I
Saad (21)	2022	ACIS	71	Apalutamide plus abiraterone	Abiraterone	32.3^b^	0(68%) 1(32%)	I

a μg/L; ng/mL.

b Interventions as treatment line I or II.

c Patients were required to have received prior treatment with docetaxel in the mCRPC setting, but response to this treatment was not necessary. —, Not Mentioned.

**Table 2:** Summary results of the trial endpoints included in the analysis.

	PROfound				NCT01972217				PROpel				ACIS			
	^a^O(n=256)	^b^A(n=131)	HR or OR(95%CI)	P	O plus A(n=71)	A(n=71)	HR(95%CI)	P	O plus A(n=399)	A(n-397)	HR or OR(95%CI)	P	^c^APA plus A(n=492)	A(n=490)	HR or OR(95%CI)	P
rPFS	5.8	3.5	0.49(0.38-0.63)^d^	<0.001	13.8	8.2	0.65(0.44-0.97)^d^	0.034	24.8	16.6	0.66(0.54-0.81)^d^	<0.0001	24.0	16.6	0.70(0.60-0.83)^d^	<0.0001
OS	17.5	14.3	0.67(0.49-0.93)^d^	—	22.7	20.9	0.91(0.60-1.38)^d^	0.66	42.1	34.7	0.81(0.67-1.00)^d^	0.0544	36.2	33.7	0.95(0.81-1.11)^d^	0.50
PFS2	—	—	—	—	23.3	18.5	0.79(0.51-1.21)^d^	0.28	—	—	0.69(0.51-0.94)^d^	—	31.8	30.2	0.92(0.78-1.08)^d^	0.31
ORR	30/138	3/67	5.93(2.01-25.40)^e^	—	9/33	12/38	0.81(0.28-2.26)^e^	0.62	—	—	—	—	109/187	86/162	—	—
PSA response	73/243	12/123	—	—	34/71	30/71	—	—	—	—	—	—	391/492	357/490	—	—
CTC conversion	41/153	7/68	—	—	15/30	13/28	—	—	—	—	—	—	—	—	—	—
Overall AEs	244/256	114/130	—	—	66/71	57/71	—	—	—	—	—	—	484/490	474/489	—	—
AEs≥3	130/256	49/130	—	—	38/71	20/71	—	—	—	—	—	—	311/490	287/489	—	—

^a^O, Olaparib; ^b^A, Abiraterone; ^c^APA, Apalutamide.

^d^Stratified proportional hazards model.

^e^Odds ratio. —, Not Mentioned.

**Table 3:** Results of indirect comparisons of olaparib, olaparib plus abiraterone and apalutamide plus abiraterone at each endpoint.

	Olaparib plus abiraterone vs. Olaparib	Apalutamide plus abiraterone vs. Olaparib	Apalutamide plus abiraterone vs. Olaparib plus abiraterone
HR (95% CI)			
rPFS	1.35 (0.99-1.84)	1.43 (1.06-1.93)	1.06 (0.83-1.35)
OS	1.24 (0.86-1.79)	1.42 (0.99-2.03)	1.14 (0.90-1.45)
PFS2	—	—	1.28 (0.95-1.73)
OR (95% CI)			
ORR	0.14 (0.03-0.68)	0.21 (0.06-0.76)	1.52 (0.50-4.62)
PSA response	0.32 (0.12-0.80)	0.36 (0.18-0.75)	1.15 (0.56-2.37)
CTC conversion	0.36 (0.09-1.39)	—	—
Overall AEs	1.14 (0.30-4.31)	0.89 (0.26-3.07)	0.79 (0.19-3.33)
AEs≥3	1.72 (0.76-3.91)	0.72 (0.43-1.18)	0.42 (0.20-0.87)

—, Not Mentioned.

### Radiographic progression-free survival

**Figure 3:**
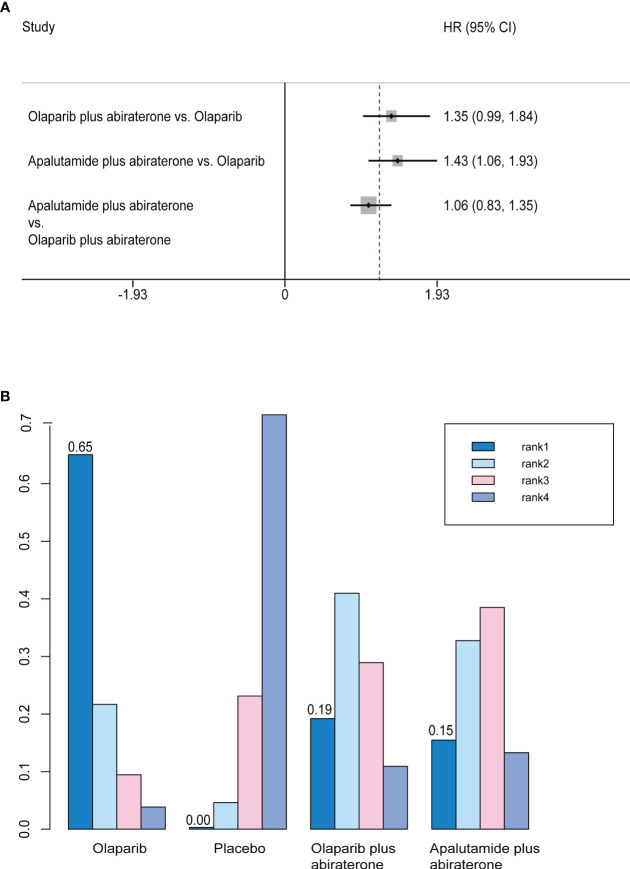
**(A)** Forest plots on the results of indirect comparisons of olaparib, olaparib plus abiraterone and apalutamide plus abiraterone for rPFS. **(B)** Rank probabilities of the three studied interventions for rPFS.

rPFS was the primary endpoint of all studies on mCRPC. In the preliminary reported data, olaparib (HR, 0.49; 95% CI, 0.38-0.63) and olaparib plus abiraterone (HR, 0.66; 95% CI, 0.55-0.79) have better rPFS compared with abiraterone. In indirect comparison, rPFS was in favor of olaparib compared with apalutamide plus abiraterone (HR, 0.70; 95% CI, 0.52-0.95). There were no significant differences between olaparib plus abiraterone and olaparib or apalutamide plus abiraterone (**Figure 3A**). Regarding rank probability, olaparib was the preferred treatment in 65.0% of the patients, followed by olaparib plus abiraterone in 19.2%, and apalutamide plus abiraterone in 15.5% (**Figure 3B**).

### Overall survival

**Figure 4:**
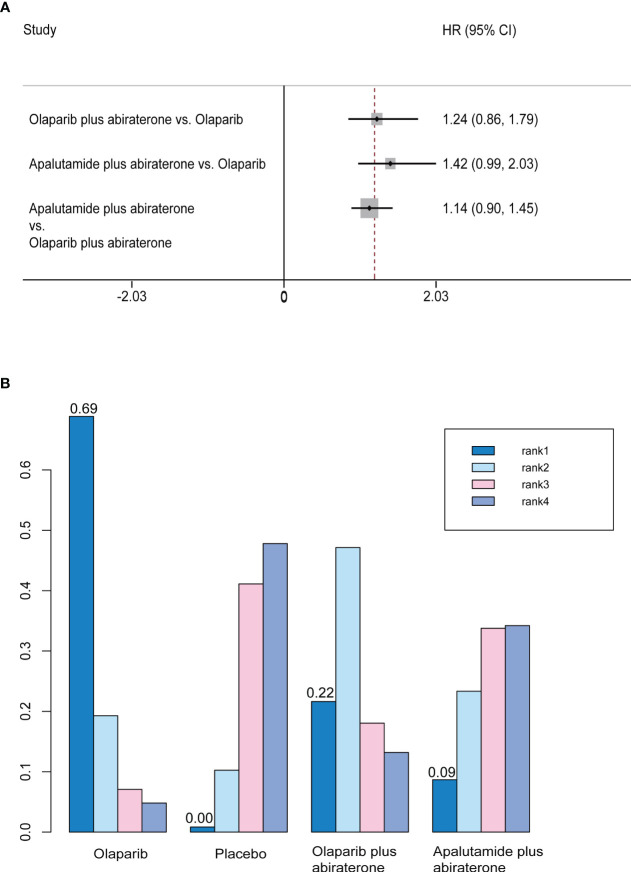
**(A)** Forest plots on the results of indirect comparisons of olaparib, olaparib plus abiraterone and apalutamide plus abiraterone for OS. **(B)** Rank probabilities of the three studied interventions for OS.

We did not find any differences between olaparib, olaparib plus abiraterone, and apalutamide plus abiraterone in indirect comparisons (**Figure 4A**). In the rank probability analysis, olaparib had a 68.9% probability of being the better choice, followed by olaparib plus abiraterone (21.6%), and apalutamide plus abiraterone (8.7%) (**Figure 4B**).

### Time to second progression-free survival

**Figure 5:**
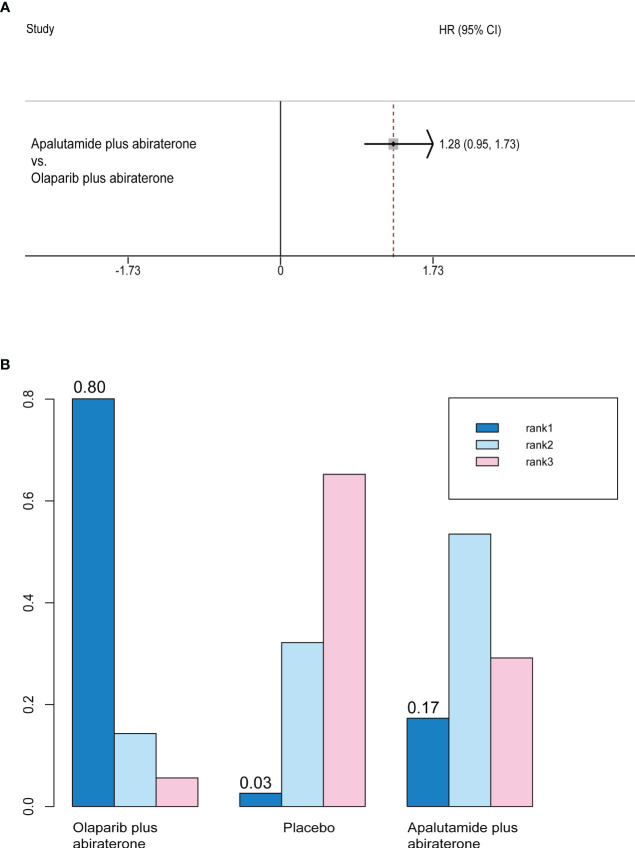
**(A)** Forest plots on the results of indirect comparisons of olaparib, olaparib plus abiraterone and apalutamide plus abiraterone for PFS2. **(B)** Rank probabilities of the three studied interventions for PFS2.

Only olaparib plus abiraterone and apalutamide plus abiraterone studies reported PFS2. There was no significant difference between the efficacy of two interventions (HR, 0.78; 95% CI, 0.58-1.06) (**Figure 5A**). Rank probability analysis demonstrated that olaparib plus abiraterone had 80.1% probability of being the preferred treatment option for prolonging PFS2 (**Figure 5B**).

### Objective response rate

We used the number of events in each arm of the included studies to calculate the OR and effect size for indirect comparisons of ORR. ORR was in favor of olaparib compared with olaparib plus abiraterone (Olaparib plus abiraterone vs. Olaparib: OR, 0.14; 95% CI, 0.03-0.68) and apalutamide plus abiraterone (Apalutamide plus abiraterone vs. Olaparib: OR, 0.21; 95% CI, 0.06-0.76). But apalutamide plus abiraterone and olaparib plus abiraterone did not significantly differ (**Table 3**). The rank probabilities of choosing apalutamide plus abiraterone and olaparib plus abiraterone as the preferred treatment options were also similar (0.7% vs. 0.8%).

### PSA response

When indirectly compared with apalutamide plus abiraterone and olaparib plus abiraterone, the results favored olaparib alone (**Table 3**). There were no significant differences between the olaparib plus abiraterone and apalutamide plus abiraterone groups. In terms of rank probability, olaparib plus abiraterone was the preferred treatment in 0.7% of the patients, followed by apalutamide plus abiraterone in 0.2%.

### Circulating tumor cell conversion

In indirect comparisons, there was no significant difference between olaparib and olaparib plus abiraterone (OR, 0.36; 95% CI, 0.09-1.39) (**Table 3**). Olaparib had a 93.3% probability of being the treatment of choice.

### Adverse events

In indirect comparisons, we did not find a significant difference in the rate of overall AEs among the three studied medications. But it was preferable to choose apalutamide plus abiraterone rather than olaparib plus abiraterone (OR, 0.42; 95% CI, 0.20-0.87) for AEs≥3 (**Table 3**). Collectively, the probability of choosing apalutamide plus abiraterone was the highest (the lowest occurrence of any AEs and AEs≥3). Secondly, the overall AEs rate of olaparib plus abiraterone was lower than olaparib monotherapy; AEs≥3 rate was reversed.

### Subgroup analysis

Subgroup analyses of rPFS and OS were available for the olaparib plus abiraterone and olaparib studies. We used HRR mutations in the subgroup analysis. In patients with mCRPC who had HRR mutations, it was confirmed no significant difference between two interventions for rPFS (HR, 1.14; 95% CI, 0.73-1.78) or OS (HR, 0.99; 95% CI, 0.60-1.61).

## Discussion

Approximately 100,000 men in the United States have non-metastatic castration-resistant prostate cancer (nmCRPC), and 86% may progress from non-metastatic CRPC to mCRPC (22). In the past decade, olaparib was the first true targeted therapy approved for prostate cancer, as a response requires the presence of DNA damage repair aberrations (23). And thanks to the results of PROpel trial, olaparib in combination of abiraterone acetate plus predisolone was approved by the FDA to treat patients with previously untreated mCRPC harboring deleterious or suspected deleterious BRCA mutations in May 2023. In studies of olaparib, olaparib plus abiraterone, and apalutamide plus abiraterone, rPFS was the primary endpoint. Preliminary data showed that all three interventions prolonged rPFS compared to abiraterone monotherapy, and all were statistically significant. The median rPFS results for all reported trials were comparable, whereas the median rPFS was longer in the intervention group than in the abiraterone group (**Table 2**). Notably, unlike the TOPARP-A study, in which the efficacy of olaparib monotherapy in patients with mCRPC was limited to patients with HRR mutations, a trial of olaparib in combination with abiraterone (NCT01972217) showed a potential clinical benefit for patients regardless of HRR mutations (13). In addition, while direct comparisons between olaparib and NAA monotherapy (enzalutamide or abiraterone) were made in the PROfound trial, there was no direct comparison between olaparib and NAA combination therapy in the current study. The inclusion of the ACIS trial for indirect comparison with olaparib, without considering the patient’s HRR mutation status, addressed this issue to some extent. Because all three therapies demonstrated similar results in rPFS, it is important to differentiate the utility of these drugs based on their differences. Our network meta-analysis indirectly demonstrated that olaparib had better rPFS than apalutamide plus abiraterone, but there seemed to be no difference between olaparib plus abiraterone and the other two interventions. To further define the options, the rank probability model in rPFS demonstrated that the preferred drug treatment was olaparib, followed by olaparib plus abiraterone and apalutamide plus abiraterone. The subgroup analysis did not reveal a difference in the rPFS benefit between olaparib and olaparib plus abiraterone in patients with HRR mutations.

The analyses of OS and PFS2 reported in each trial are immature. The OS reported in the preliminary studies did not attain statistical significance. Possible causes are that the patient received subsequent life-prolonging treatments, which did not include the three interventions we studied. In addition, uncertainty regarding the optimal sequencing of the combination of two active therapies in mCRPC has resulted in no statistically significant data on the OS benefit, and clinical trials to date have confirmed this view (20). Although there were no significant differences in the indirect comparisons, the magnitude and direction of PFS2 data were consistent with the primary rPFS analysis.

The development of response biomarkers to rapidly identify drug-resistant diseases and guide early therapeutic conversion remains an unmet clinical need. The value of CTC as prognostic indicators for advanced prostate cancer has been well described (24, 25). These data indicate that CTC progression with low baseline CTC counts (<5) during the first 12 weeks of chemotherapy or endocrine therapy can identify patients who will not benefit from treatment. This could guide patient response assessment during the first 12 weeks of treatment, identify early disease progression, and serve as a biomarker of efficacy in clinical trials (26). In olaparib plus abiraterone trial, CTC conversion rates were similar in both arms; whereas in the olaparib alone trial the intervention arm was higher than the comparator arm (27% vs. 10%). In the olaparib plus abiraterone and apalutamide plus abiraterone trials, the PSA response rates were similar in both arms, which might be a consequence of the potency of abiraterone and its cytostatic mode of action (27). Thus, the difference in rPFS between the two groups may be due to an increased proportion of patients with stable disease and a longer duration of response in the combination arm rather than an increase in the number of patients with a complete response.

Adverse effects and quality-of-life aspects of treatment options are important factors in the decision-making process, as cancer presentation and long-term survival rates were similar in patients treated with the three interventions. The four most common AEs in patients treated with olaparib were anemia, nausea, fatigue/asthenia, and decreased appetite, consistent with the results of a meta-analysis of the risk of AEs in studies on olaparib in other tumor types (28). The most common AE reported in the phase II clinical trials of olaparib plus abiraterone was nausea. The most common ≥3 AE, however, was anemia, which was also consistent with the results reported by PROpel. As we were unable to obtain a detailed number of each adverse event in PROpel in the trial of olaparib plus abiraterone, the analysis of the AEs results in our study was based primarily on the results of a phase II clinical trial. Thus, insufficient patient numbers may account for the difference in the choice of olaparib, with or without abiraterone, for AEs and ≥3 AEs. A further study of PROfound found an increased risk of four common adverse events not significantly associated with prior taxane therapy or BRCA alterations (29). The incidence of these four AEs peaks in the first two months of treatment and can be controlled by dose adjustment and supportive therapy without stopping treatment, which suggests that the management of AEs should be accompanied by a focus on the clinical benefit of patients.

CAPTURE trial reported that BRCA patients, regardless of somatic/germline origin, exhibited considerably lower rPFS, PFS2, and OS, as well as significantly worse PFS2 and OS than non-BRCA patients in the HRR subgroup. These poor results were not attributable to the BRCA 1/2 subgroup receiving fewer active mCRPC therapies. And these situations could be avoided by adding PARPi (30). PARPi other than Olapali are also worth attention. The TRITON3 trial compared the efficacy of rucaparib monotherapy to the doctor’s choice of chemotherapy, abiraterone, or enzalutamide in chemotherapy-naive patients with BRCA or ATM mutations (31). In the experimental group, rPFS considerably improved (median 10.2 vs. 6.4 months; HR, 0.61; 95% CI 0.47-0.80; P = 0.0003). In the subgroup analysis, rucaparib maintained its rPFS advantage in the BRCA-mutated cohort, but there was no difference between the two treatment arms in patients with ATM mutations (median rPFS 8.1 vs. 6.8 months; HR, 0.97; 95% CI 0.59–1.52; P = 0.84). In another phase III trial TALAPRO-2, patients were randomized to receive enzalutamide plus talazoparib or enzalutamide plus placebo; all patients benefited from combination therapy regardless of HRR status, but the benefit was more pronounced in patients with HRR mutations (rPFS 27.9 VS. 16.4; HR, 0.46; 95% CI 0.3–0.7; P < 0.001) (32). Another FDA-approved PARPi combination therapy was based on MAGNITUDE; this trial, which included the largest BRCA1/2 cohort to date in first-line mCRPC, demonstrated that niraparib plus abiraterone significantly improved rPFS in BRCA1/2 patients compared with abiraterone plus placebo (19.5 vs. 10.9 months; HR, 0.55; P= 0.0007) (33). Consistent with PARPi monotherapy, the combination regimen was most beneficial for BRCA mutations patients, followed by HRR mutations patients, and most unfavorable for HRR proficient mCRPC patients. These results further support the importance of screening germline and somatic BRCA1/2 alterations for a more precise patients care (30).The assumption that PARPi binding to NAA is beneficial regardless of BRCA mutation status is being challenged based on recent final overall survival data from the PROpel trial, as well as interim analyses of the TALAPRO-2 and MAGNITUDE trials. The discrepancy between PROpel results and the actual FDA drug approval also highlights that the need to explore whether patients with non-HRR mutations benefit from the combination of PARPi with NAA (34). In addition, given the inconsistent inclusion and design criteria of these PARPi trials, which do not allow for direct comparison of results, the selection and sequencing of these drugs will be increasingly challenging in the clinic.

The findings of this review should be interpreted within the context of its limitations. First, our analysis relied on indirect comparisons, which have inherent limitations and can be misleading if there are significant differences in baseline study populations between studies. Second, we chose HR and OR; different utility measures provide explanations for drug efficacy that must be viewed with caution and validated further. Additionally, the included studies did not report the type of HRR-mutated genes, and the difference between PARP inhibitors combined with or without NAA in patients with HRR mutations needs to be further explored. As the analyses of AEs in the olaparib plus abiraterone trials were insufficient, we only studied overall AEs and ≥3 AEs and did not perform further data analysis on the four common AEs caused by PARP inhibitors. Despite these limitations, we carefully selected evidence, and the screened studies were high-quality randomized controlled trials with similar patient-selection criteria. We also demonstrated the utility of PARP inhibitors in prolonging the survival of patients with high-risk prostate cancer. We also provided a basis for clinicians to target specific endpoints.

## Conclusion

We found that all three interventions including olaparib, olaparib plus abiraterone and apalutamide plus abiraterone significantly improved rPFS. In the absence of found differences in efficacy between interventions, olaparib might be the optimal choice. Apalutamide plus abiraterone might be taken into consideration as a substitute for olaparib and olaparib plus abiraterone when AEs limited their use.

## Data availability statement

The datasets presented in this study can be found in online repositories. The names of the repository/repositories and accession number(s) can be found in the article/**Supplementary Material**.

## Author contributions

XC and YP designed the study and analyzed the data. QW and CR revised the images. ML, XH, and LX performed the literature search and collected data for the manuscript. XL revised the manuscript. All authors contributed to the article and approved the submitted version.
