# Supplementary material for: Comparative efficacy of olaparib in combination with or without novel antiandrogens for treating metastatic castration-resistant prostate cancer
Source: Front Endocrinol (Lausanne). 2023 Oct 31;14:1225033. doi: 10.3389/fendo.2023.1225033 (PMC10644304; doi:10.3389/fendo.2023.1225033)
Supplement: Supplementary file 1 [file DataSheet_1.docx]

**Supplementary data. Search strategy**

**Pubmed**

#1 “olaparib”[MeSH Terms] OR AZD 2281[All Fields] OR AZD2281[All Fields] OR AZD-2281[All Fields] OR AZD221[All Fields] OR Lynparza[All Fields]

#2 “Abiraterone Acetate”[MeSH Terms] OR 17-(3-pyridyl)-5,16-androstadien-3beta-acetate[All Fields] OR Zytiga[All Fields] OR CB 7630[All Fields] OR CB-7630[All Fields] OR CB7630[All Fields]

#3 “apalutamide”[MeSH Terms] OR ARN-509[All Fields] OR Erleada[All Fields]

#4 “enzalutamide”[MeSH Terms] OR 4-(3-(4-cyano-3-(trifluoromethyl)phenyl)-5,5-dimethyl-4-oxo-2-thioxo-1-imidazolidinyl)-2-fluoro-N-methyl-benzamide[All Fields] OR enzalutamide D3[All Fields] OR HC-1119[All Fields] OR HC 1119[All Fields] OR 4-(3-(4-cyano-3-(trifluoromethyl)phenyl)-5,5-dimethyl-4-oxo-2-thioxo-1-imidazolidinyl)-2-fluoro-N-(methyl-d3)benzamide[All Fields] OR Xtandi[All Fields] OR MDV 3100[All Fields] OR MDV3100[All Fields] OR MDV-3100[All Fields]

#5 “darolutamide”[MeSH Terms] OR Nubeqa[All Fields] OR ORM-16497[All Fields] OR ODM-201[All Fields] OR ORM-16555[All Fields]

#6 “Prostatic Neoplasms”[MeSH Terms] OR Prostate Neoplasms[All Fields] OR Prostate Neoplasm[All Fields] OR Prostatic Neoplasm[All Fields] OR Prostate Cancer[All Fields] OR Prostate Cancers[All Fields] OR Prostatic Cancer[All Fields] OR Prostatic Cancers[All Fields]

#7 ((randomized controlled trial[pt] OR controlled clinical trial[pt] OR randomized[tiab] OR randomised[tiab] OR placebo[tiab] OR drug therapy[sh] OR randomly[tiab] OR trial[tiab] OR groups[tiab]) NOT (animals[mh] NOT humans[mh]))

#8 #1 OR #2 OR #3 OR #4 OR #5

#9 #6 AND #7 AND #8

**EMBASE**

1 Randomized controlled trial/

2 Controlled clinical study/

3 Random$.ti,ab.

4 randomization/

5 intermethod comparison/

6 placebo.ti,ab.

7 (compare or compared or comparison).ti.

8 (open adj label).ti,ab.

9 ((double or single or doubly or singly) adj (blind or blinded or blindly)).ti,ab.

10 double blind procedure/

11 parallel group$1.ti,ab.

12 crossover or cross over).ti,ab.

13 ((assign$ or match or matched or allocation) adj5 (alternate or group$1 or intervention$1 or patient$1 or subject$1 or participant$1)).ti,ab.

14 (assigned or allocated).ti,ab.

15 (controlled adj7 (study or design or trial)).ti,ab.

16 (volunteer or volunteers).ti,ab.

17 trial.ti.

18 or/1-17

19 exp prostate/

20 exp prostate disease/

21 (19 or 20) and exp neoplasm/

22 exp prostate tumor/

23 exp prostate cancer/

24 exp prostate carcinoma/

25 (prostate$ adj5 (neoplas$ or cancer$ or carcin$ or tumo$ or metasta$ or malig$)).ti,ab.

26 (metastatic adj5 (prostate adj5 (neoplasm$ or cancer$ or carcinoma$ or tumo?r$))).ti,ab.

27 or/21-26

28 exp nicotinamide adenine dinucleotide adenosine diphosphate ribosyltransferase/

29 exp nicotinamide adenine dinucleotide adenosine diphosphate ribosyltransferase inhibitor/

30 poly ADP ribose polymerase inhibitor$.mp.

31 (poly adj5 ADP adj5 ribose adj5 polymerase adj5 inhibit$).mp.

32 ((PARP adj5 inhibit$) or PARP inhibit$).mp.

33 exp olaparib/

34 (olaprib or AZD-2281 or AZD 2281).mp.

35 or/28-34

36 exp abiraterone/

37 (abiraterone or CB-7630 or CB 7630).mp.

38 exp apalutamide/

39 (apalutamide or ARN-509).mp.

40 exp enzalutamide/

41 (enzalutamide or MDV 3100 or MDV-3100).mp.

42 exp darolutamide/

43 (darolutamide or ORM-16497 or ODM-201).mp.

44 or/36-43

45 35 or 44

46 18 and 27 and 45

47 limit 46 to (human and (conference abstracts or embase))

48 limit 47 to yr=”2010-Current”

**CENTRAL**

#1 MeSH descriptor: [Prostatic Neoplasms] explode all trees
#2 metasta* near prostate near cancer* or metastatic prostate cancer*
#3 metasta* near prostate near neoplasm* or metastatic prostate neoplasm*
#4 metasta* near prostate near carcinom* or metastatic prostate carcinom*
#5 metasta* near prostate near tumour* or metastatic prostate tumour*
#6 metasta* near prostate near tumor* or metastatic prostate tumor*
#7 #1 or #2 or #3 or #4 or #5 or #6
#8 MeSH descriptor: [Poly(ADP-ribose) Polymerases] explode all trees
#9 ‘Poly (ADP-ribose) Polymerase inhibitor’ or Poly (ADP-ribose) near Polymerase* near inhibitor
#10 PARP* inhibit* or PARP* near inhibit*
#11 olaparib or AZD-2281

#12 abiraterone or CB-7630

#13 apalutamide or ARN-509

#14 enzalutamide or MDV-3100

#15 darolutamide or ORM-16497
#16 #8 or #9 or #10 or #11 #12 or #13 or #14 or #15
#17 #7 and #16

**ASCO meeting library**

#1 olaparib

#2 metastatic prostate cancer

#3 #1 and #2

All databases last searched on March 25, 2023.
